# The Impact of the Lactation Period Gut Microbiota of Two Different Beef Cattle Breeds on Spring-Born Calves

**DOI:** 10.3390/ani15020197

**Published:** 2025-01-13

**Authors:** Changbo Chen, Yuzhu Sha, Xiaoqiang Zhang, Pingle Lu, Jianyuan Gao, Ting Jiao, Shengguo Zhao

**Affiliations:** 1 College of Animal Science and Technology, Gansu Agricultural University, Lanzhou 730070, China; changbochen1996@163.com (C.C.);; 2 Provincial R&D Institute of Ruminants in Gansu, Lanzhou 730070, China; 3 Gansu Dongniu Science and Technology Innovation Development Center, Jingchuan 744300, China; 4 Red Cattle Industry Service Center, Jingchuan County Animal Husbandry and Veterinary Center, Jingchuan 744300, China; 5 School of Bioengineering, Aksu Vocational and Technical College, Aksu 843000, China; 6 College of Grassland Science, Gansu Agricultural University, Lanzhou 730070, China

**Keywords:** Pingliang red cattle, Simmental cattle, intestinal microbiota, immune parameters, milk quality

## Abstract

To investigate the impact of maternal microbiota during lactation in different beef cattle breeds on their own immune levels, milk quality, and the growth and development of their offspring, this study measured the immune parameters, intestinal microbiota diversity, and milk quality of Pingliang red cattle and Simmental cattle, and performed a correlation analysis with the growth and development of their offspring. Our study showed that during lactation, Pingliang red cattle had significantly higher IL-6 levels than Simmental cattle, while the latter exhibited higher levels of immune factors such as IgG, IgA, IgM, IL-1β, and TNFα. The analysis of the intestinal microbiota of lactating cows found that Pingliang red cattle were rich in *Bacteroidetes* and *Fibrobacteres*, while Simmental cattle had a higher proportion of Actinobacteria. This difference may be related to the different adaptation strategies in energy metabolism and immune regulation between the two breeds. In addition, the analysis of milk quality between different beef cattle breeds revealed significant differences in protein, acidity, milk fat, and total solids. The correlation analysis found that *Alistipes_communis*, *Firmicutes_bacterium_CAG_110*, *Alistipes_communis*, *Paludibacter_propionicigenes*, *Alistipes_sp._58_9_plus*, and *Bacteroidales_bacterium_55_9* were associated with both milk quality and the growth of offspring calves, including body weight, body length, chest girth, and cannon circumference. In conclusion, this study provides new insights into the intestinal microbiota, immune characteristics, and their effects on offspring health in different beef cattle breeds, and has important implications for breed selection and management strategies in the livestock industry.

## Simple Summary

This study investigates the impact of gut microbiota during lactation in two beef cattle breeds, Pingliang red cattle and Simmental cattle, on their immune levels, milk quality, and the growth of their offspring. Significant differences were found between the breeds: Pingliang red cattle had higher levels of IL-6, while Simmental cattle had higher levels of antibodies like IgG and IgA. The gut microbiota composition also varied, affecting milk quality and calf growth. Pingliang red cattle had more Bacteroidetes, which aids fiber digestion, whereas Simmental cattle had higher Actinobacteria levels. Milk quality differences, such as higher fat content in Simmental cattle, influenced the growth rate of calves. Overall, this study highlights how gut microbiota and milk composition during lactation influence calf health and development, providing insights for breed selection and management strategies in the livestock industry.

## 1. Introduction

The beef cattle industry holds an important position in global livestock farming and is one of the primary sources of high-quality animal protein. As living standards rise, the demand for beef and the expectations for its quality continue to grow [1]. In China, Pingliang red cattle and Simmental cattle are two representative beef cattle breeds. Pingliang red cattle have gained widespread attention for their strong adaptability and delicious meat quality [2]. Simmental cattle are renowned for their rapid growth rate and high meat production performance [3,4]. However, these two breeds exhibit significant differences in production performance, milk quality, and immune function, which may affect the growth and development of their offspring. The gut microbiota plays a key role in the nutritional metabolism, immune regulation, and maintenance of health in animals [5]. The gut microbiota not only participates in the digestion of feed and the absorption of nutrients but also influences the host’s immune function through interactions with the host immune system [6,7]. Studies suggest that the composition and function of the maternal gut microbiota may have a significant impact on mammary gland health, milk composition, and the development and immune system maturation of offspring [8,9]. The lactation period is a critical stage for the physiological and immune system development of both the adult cow and the calf. The cow provides essential nutrients and immune protection to the calf through milk [10]. The nutritional components and bioactive substances in milk (immunoglobulins, lactoferrin, etc.) play a crucial role in the growth and immune function development of calves [10]. The differences in milk quality among different cow breeds may lead to variations in calf growth performance and health status [11]. However, research on the correlation between the gut microbiota, immune parameters, milk quality, and calf growth and development across different cow breeds remains limited. Research has begun to focus on the impact of the cow’s gut microbiota on milk quality and immune function. Studies have shown that the diversity and stability of the gut microbiota are closely related to the cow’s immune status, affecting the levels of immune-active substances in milk [12]. For example, specific beneficial microbiota can promote the secretion of immunoglobulins in cows, thereby improving milk quality [13]. In addition, milk quality has a significant impact on calf growth, development, and immune system maturation [14,15]. High-quality milk helps to increase the daily weight gain of calves and enhances their disease resistance [16]. During the early growth stages of calves, the nutritional components and bioactive substances in cow’s milk are crucial for the colonization of the calf’s gut microbiota and the development of its immune system [17]. However, there is a lack of systematic comparative studies on these two important beef cattle breeds, Pingliang red cattle and Simmental cattle. This limits our understanding of the differences in production performance and health status among beef cattle breeds, and hinders the implementation of breed improvement and precision feeding management.

This study aims to analyze the immune parameters, intestinal microbiota, and milk quality between Pingliang red cattle and Simmental cattle cows during lactation. It investigates the impact of intestinal microbiota from different breeds of cows on immune levels and milk quality, exploring the underlying mechanisms. The study also examines how milk quality and maternal intestinal microbiota affect the growth and development of calves before weaning, revealing the pathways through which cows influence calf growth performance. The results of this study provide scientific guidance for the health management and feeding practices of different beef cattle breeds during lactation.

## 2. Materials and Methods

### 2.1. Experimental Design and Sample Collection

All animal experiments were reviewed and approved by the Animal Committee of Gansu Agricultural University. This study was conducted at the Gansu Dongniu Science and Technology Innovation Center in Jingchuan County, Gansu Province, China. After synchronizing estrus and performing artificial insemination on the non-pregnant Pingliang red cattle and Simmental cattle, and after calving, 3 male calves and 3 female calves were randomly selected from each of their respective herds, and the corresponding dams were designated as subjects for the study. Throughout the entire experimental period, all cows were allowed food under uniform feeding and management conditions. To eliminate technical biases and improve the resolution of changes related to animal physiological status, all cows selected for this study were from the same farm, with a consistent diet formulation throughout the experimental period, and the entire study process was strictly standardized. Blood samples and intestinal feces were collected from the cows during pregnancy and lactation (Lp, 90 days postpartum), as well as blood samples and intestinal feces from their offspring calves. Calf weight and body measurements were recorded at 0 days and 180 days, and milk samples were manually collected for milk quality analysis. A 3 mL blood sample (non-anticoagulant tube) was collected via the jugular vein, centrifuged at 3000 rpm for 15 min to separate the serum, which was then transferred to cryopreservation tubes. The collected feces were transferred to 10 mL cryopreservation tubes. The cryopreservation tubes were placed in liquid nitrogen tanks, transported back to the laboratory, and stored at −80 °C. Metagenomic sequencing was performed on the intestinal feces samples from both the cows and the calves. Immune indices of lactating cows and their offspring calves were measured.

### 2.2. Measurement of Immune Parameters

Immune parameters in blood were assessed using various bovine ELISA kits: IgA (F72006-A), IgG (F3902-A), IgM (F72008-A), IL-1β (F3895-A), IL-6 (F3894-A), and TNF-α (F72110-A), all obtained from Fankewi Company, Shanghai, China. The assays were conducted according to the manufacturer’s protocols. Measurements were taken using a Thermo 3020 microplate reader. The procedure included adding the samples and standards to the ELISA plate, followed by incubation at 37 °C for 30 min and five washes. The enzyme-labeled reagent was then added, incubated at 37 °C for another 30 min, and washed five more times. Afterward, substrates A and B were introduced to allow color development at 37 °C for 10 min, followed by the addition of a stop solution. The optical density (OD) at 450 nm was measured, with the blank set to zero. OD values were read within 15 min, and the concentration was calculated accordingly.

### 2.3. Metagenome Sequencing and Bioinformatics Analysis

Bacterial DNA was extracted from fecal samples using the TGuide S96 Magnetic Stool DNA Kit (Beijing, China), following the manufacturer’s instructions. The genomic DNA was enzymatically fragmented to generate appropriate-sized fragments for analysis. These fragments underwent end repair, adaptor ligation, amplification, and purification to generate a usable library. Fragment quality was evaluated with the Qsep-400 system, and sequencing was performed on the Illumina NovaSeq6000 platform (Illumina Inc., San Diego, CA, USA). Raw sequencing tags were processed and cleaned using fastp (version 0.23.1) [18], and then aligned to the host genome using bowtie2 (version 2.2.4) [19] to remove host contamination and generate clean tags of high quality. Macrogenome assembly was carried out using MEGAHIT (version 1.1.2) [20], with contig sequences shorter than 300 bp filtered out, and assembly quality was assessed using QUAST (version 2.3) software [21]. Redundancy was reduced using MMseqs2 (https://github.com/soedinglab/mmseqs2, Version 12-113e3, accessed on 2 October 2023) [22], applying thresholds of 95% similarity and 90% coverage. The annotation of carbohydrate-active enzymes was performed by comparing the protein sequences of non-redundant genes to the CAZy [23] database. Finally, representative sequences from the non-redundant gene catalog were aligned with the Nr [24] and KEGG [25] (Kyoto Encyclopedia of Genes and Genomes) databases using BLASTP (the expected e-value is 10−5) to obtain detailed annotation information.

### 2.4. Data Statistics and Analysis

PCoA analysis, α-diversity analysis, and LEfSe analysis of the experimental samples were performed using the Biocloud platform (https://international.biocloud.net, accessed on 6 October 2024). Additionally, Spearman correlation tests were used for the correlation analysis, with a significance level of *p* < 0.05. Data analysis was performed using IBM SPSS Statistics 25.0 software, and independent sample *t*-tests were used to analyze immune parameters and milk quality levels.

## 3. Results

### 3.1. Differences in Blood Immune Parameters During Lactation Between the Two Beef Cattle Breeds

**Figure 1:**
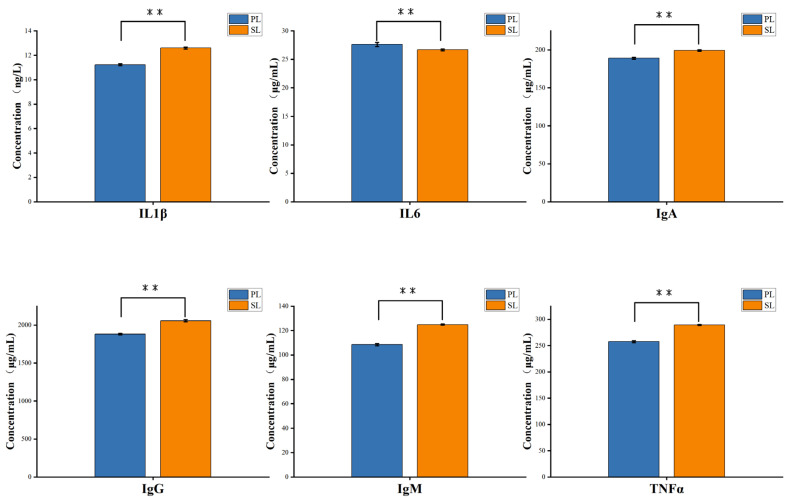
Differences in immune parameters between Pingliang red cattle and Simmental cattle. Note: ** represents highly significant difference (*p* < 0.01).PL represents Pingliang red cattle during the lactation period, and SL represents Simmental cattle during the lactation period.

As shown in Figure 1, there are highly significant differences (*p* < 0.01) in blood immune parameters between the two breeds. Pingliang red cattle had significantly higher levels of Interleukin-6 (IL-6) compared to Simmental cattle, while the levels of IgA, IgG, IgM, IL-1β, and TNF-α were significantly lower (*p* < 0.01) in Pingliang red cattle.

### 3.2. Comparison of Microbiota During Lactation Between the Two Beef Cattle Breeds

Raw data were obtained from the metagenomic sequencing of the gut contents of Pingliang red cattle and Simmental cattle. After filtering out low-quality sequences, 71,438,888,071 and 80,962,460,832 sequences were obtained for Pingliang red cattle and Simmental cattle, respectively. Finally, 468,818,806 and 533,295,472 sequences were obtained for Pingliang red cattle and Simmental cattle, respectively.

**Figure 2:**
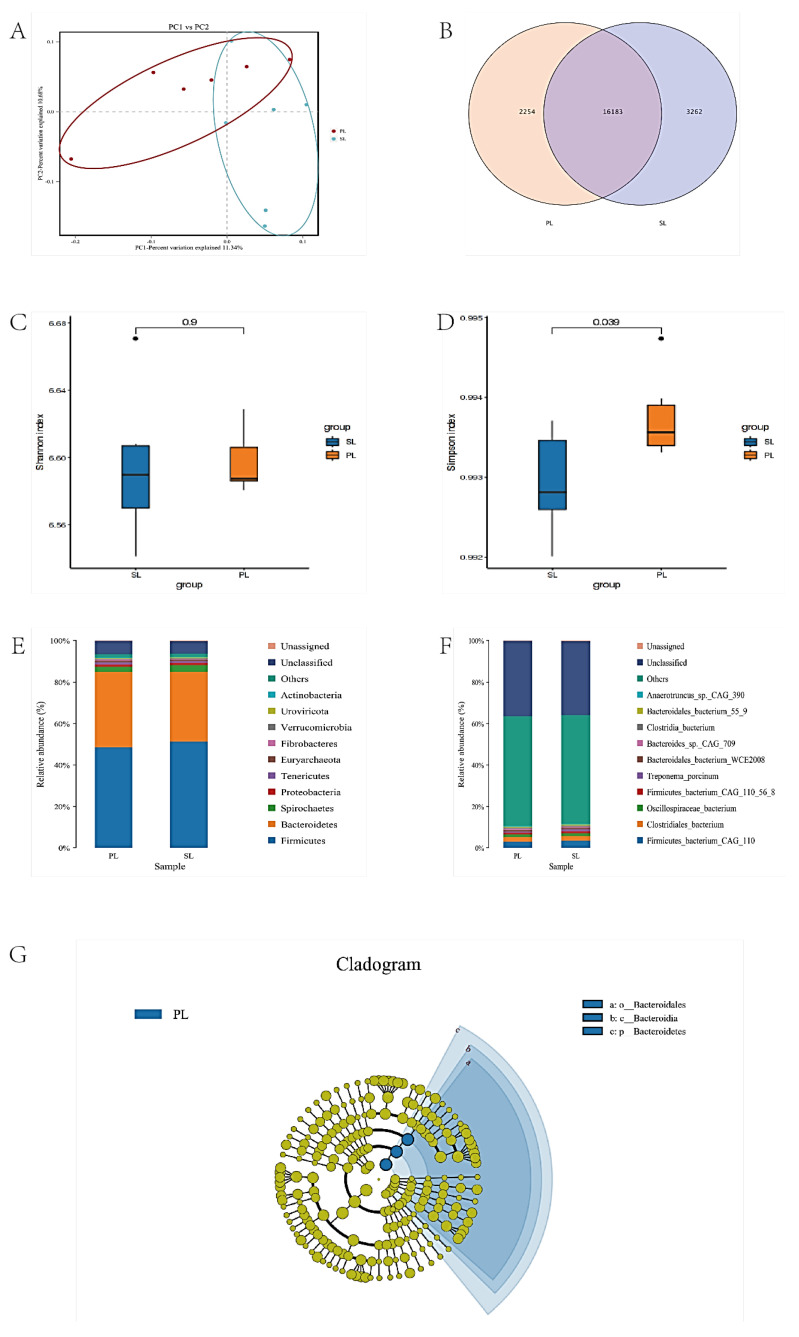
Comparison of microbiota during lactation between the two beef cattle breeds. PCoA analysis (**A**). OTU Wayne diagram (**B**). Microbial diversity indicators, Shannon and Simpson indices of Pingliang red cattle and Simmental cattle (**C**,**D**). Microbial composition at the level of phyla and species (**E**,**F**). LEfSe analysis (**G**). Note: PL represents Pingliang red cattle during the lactation period, and SL represents Simmental cattle during the lactation period.

PCoA analysis revealed differences in the gut microbiota between Pingliang red cattle and Simmental cattle during the lactation phase (Figure 2A). At the species level, a total of 18,437 bacterial species were detected in Pingliang red cattle, with 2254 species being unique; in Simmental cattle, 19,445 species were detected, with 3262 species being unique; the two breeds shared 16,183 species. This indicates that the gut microbiota of the two breeds shares certain similarities, but also exhibits significant differences.

α-diversity analysis showed no significant difference in Shannon index between the two breeds (*p* > 0.05) (Figure 2C). The Simpson index of Pingliang red cattle was significantly higher than that of Simmental cattle (*p* < 0.05) (Figure 2D).

Microbial community composition analysis showed that at the phylum level, *Firmicutes* and *Bacteroidetes* were the dominant phyla in all groups (Figure 2E). *Bacteroidetes* and *Fibrobacteres* in Pingliang red cattle were significantly higher than those in Simmental cattle, while Simmental cattle had significantly higher levels of Actinobacteria than Pingliang red cattle (*p* < 0.05) (Figure 2F). No significant differences were observed between the two breeds at the species level (*p* > 0.05), and *Firmicutes_bacterium_CAG_110* and *Clostridiales_bacterium* were the dominant species in all groups. LEfSe analysis revealed that the unique microbiota of Pingliang red cattle during lactation consisted of *o_Bacteroidales*, *c_Bacteroidia*, and *p_Bacteroidetes* (Figure 2G).

KEGG functional annotation and enrichment analysis revealed 395 differential metabolic pathways shared between Pingliang red cattleand Simmental cattle (Supplementary Table S1). Key pathways with differences include K00899 (galactose metabolism), K01000 (glucose-6-phosphate isomerase in glycolysis/gluconeogenesis), K07118 (aldehyde dehydrogenase), K03474 (ABC transporter family), and K00346 (nitrate reductase), among others. These differences primarily involve enzymes, transport proteins, receptors, and other related factors.

CAZy carbohydrate-active enzyme annotation analysis revealed 31 differential metabolic pathways shared between Pingliang red cattle and Simmental cattle (Supplementary Table S2). The major pathways include CBM10 (carbohydrate-binding module), PL42 (polysaccharide lyase), GT2 (glycosyltransferase), and others. These pathways mainly encompass glycoside hydrolases (GHs), glycosyltransferases (GTs), polysaccharide lyases (PLs), carbohydrate-binding modules (CBMs), and carbohydrate esterases (CEs), among others.

### 3.3. Differences in Milk Quality During Lactation Between the Two Beef Cattle Breeds

**Figure 3:**
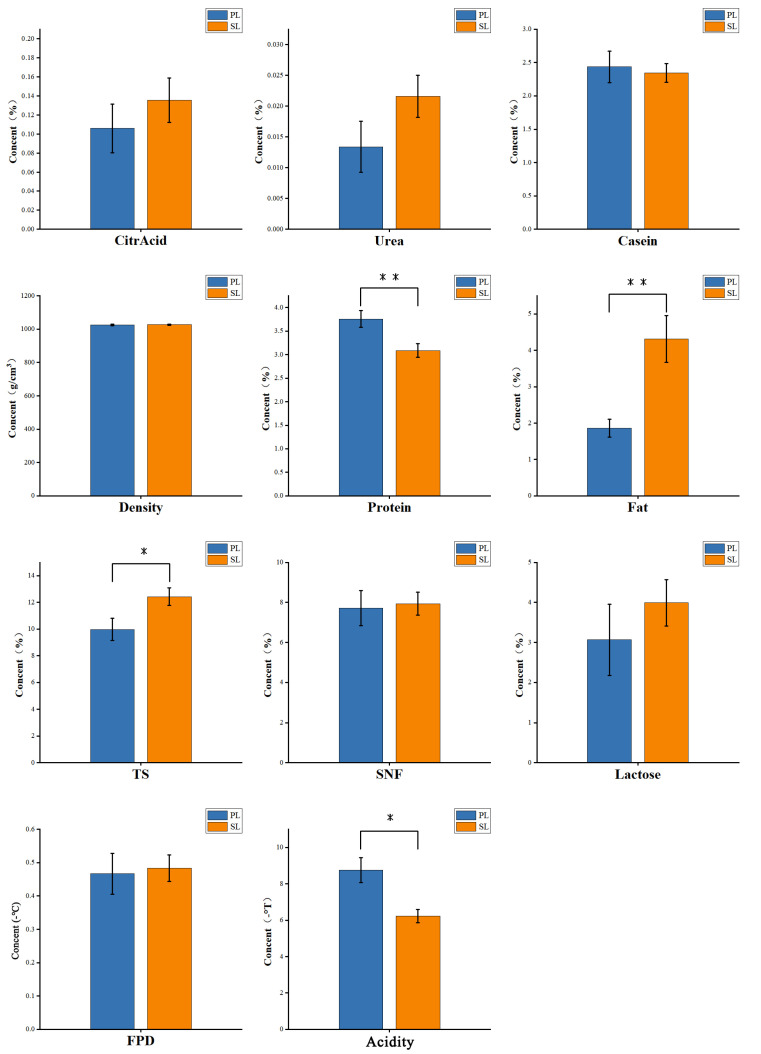
Differences in milk quality during lactation between the two beef cattle breeds. Note: * represents significant difference (*p* < 0.05); ** represents highly significant difference (*p* < 0.01). PL represents Pingliang red cattle during the lactation period, and SL represents Simmental cattle during the lactation period.

As shown in Figure 3, the analysis of the milk during lactation from the two beef cattle breeds revealed that Pingliang red cattle milk had significantly higher protein and acidity levels compared to Simmental cattle milk (*p* < 0.05). Meanwhile, Simmental cattle milk had significantly higher levels of milk fat and total solids compared to Pingliang red cattle milk (*p* < 0.05). No significant differences were observed between the two types of milk for the remaining indicators (*p* > 0.05).

### 3.4. The Impact of Lactation Microbiota on Maternal Immune Status and Milk Quality

**Figure 4:**
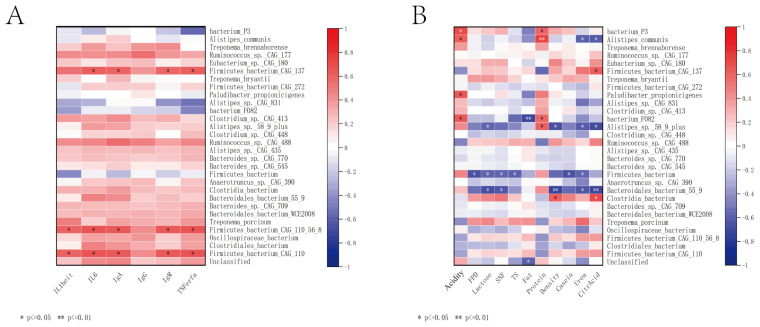
Correlation analysis of lactation microbiota with maternal immune status and milk quality. Correlation heatmap between microbiota and maternal immune status (**A**). Correlation heatmap between microbiota and milk quality (**B**).

At the species level, the lactating cows’ gut microbiota, such as *Firmicutes_bacterium_CAG_137*, showed a significant positive correlation with IL6, IgA, IgM, and TNFα (*p* < 0.05). *Firmicutes_bacterium_CAG_110_56_86* and *Firmicutes_bacterium_CAG_110* were significantly positively correlated with ILβ, IL6, IgA, IgM, and TNFα (*p* < 0.05). *Bacterium_P3* was significantly positively correlated with acidity and protein (*p* < 0.05). *Alistipes_communis* showed a significant positive correlation with acidity (*p* < 0.05), a highly significant positive correlation with protein (*p* < 0.01), and a significant negative correlation with urea and citric acid (*p* < 0.05). Firmicutes_bacterium_CAG_137 was significantly positively correlated with citric acid (*p* < 0.05). *Paludibacter_propionicigenes* was significantly positively correlated with acidity (*p* < 0.05). *Bacterium_F082* was significantly positively correlated with acidity and protein (*p* < 0.05), and highly significantly negatively correlated with Fat (*p* < 0.01). *Alistipes_sp._58_9_plus* was significantly positively correlated with protein (*p* < 0.05) and significantly negatively correlated with lactose, density, urea, and citric acid (*p* < 0.05). *Firmicutes_bacterium* was significantly negatively correlated with FPD, lactose, SNF, casein, and citric acid (*p* < 0.05). *Bacteroidales_bacterium_55_9* was significantly negatively correlated with lactose, SNF, and urea (*p* < 0.05), and highly significantly negatively correlated with density and citric acid (*p* < 0.01). *Clostridia_bacterium* was significantly positively correlated with density and citric acid (*p* < 0.05) (see Figure 4).

### 3.5. The Impact of Milk Quality During Lactation on the Pre-Weaning Growth and Development of Calves

**Figure 5:**
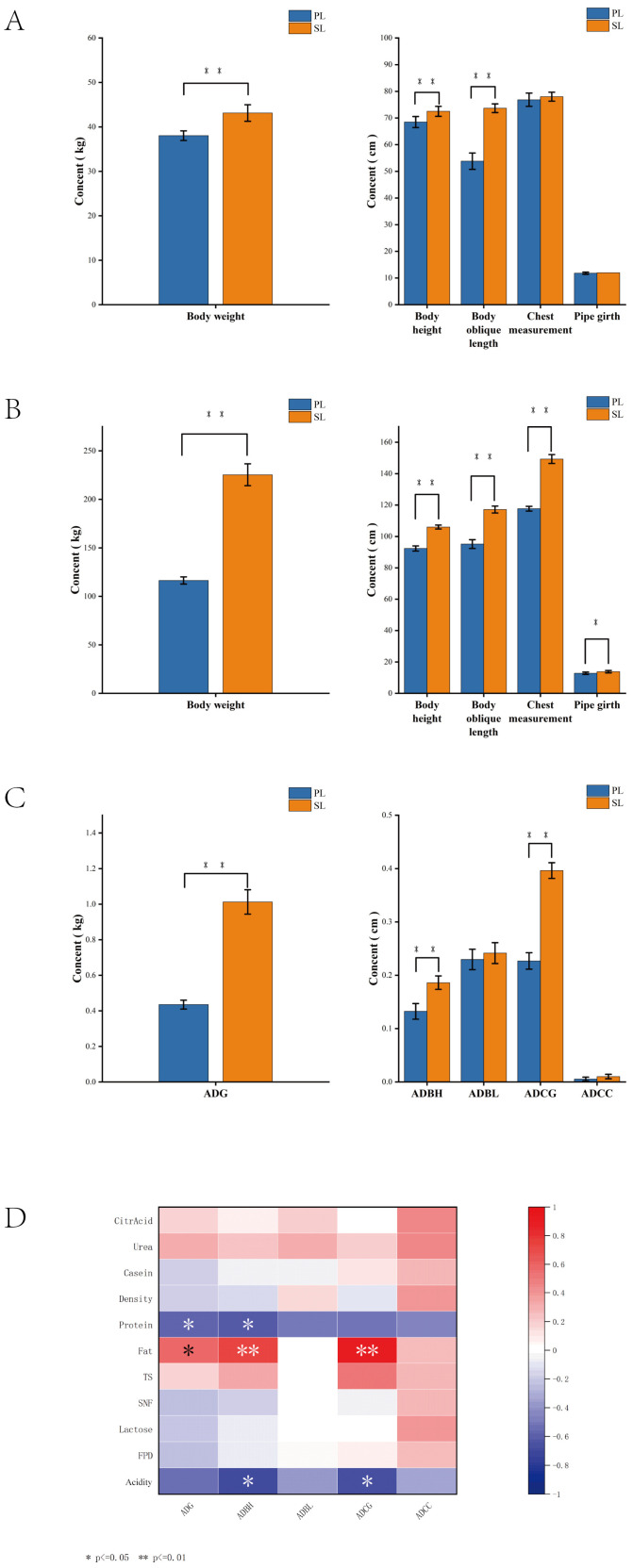
The impact of milk quality during lactation on the growth and development of calves before weaning. Comparison of weight and body measurements of Pingliang red cattle and Simmental cattle calves at 0 d and 180 d (**A**,**B**). The average daily growth of weight and body measurements during the growing period (**C**). Heatmap of the correlation between the average daily growth of weight and body circumference and milk quality (**D**). Note: * represents significant difference (*p* < 0.05); ** represents highly significant difference (*p* < 0.01). PL represents Pingliang red cattle during the lactation period, and SL represents Simmental cattle during the lactation period.

At day 0, Simmental cattle calves had significantly higher weight, body height, and body length compared to Pingliang red cattle calves (*p* < 0.01) (Figure 5A). At day 180, Simmental cattle calves had significantly higher weight, body height, body length, and chest girth (*p* < 0.01), and cannon circumference was significantly higher than that of Pingliang red cattle calves (*p* < 0.05) (Figure 5B). The average daily gain (ADG), average daily body height gain (ADBH), and average daily chest girth gain (ADCG) of Simmental cattle calves were significantly higher than those of Pingliang red cattle calves (*p* < 0.01) (Figure 5C).

Milk quality during lactation is correlated with the growth and development of calves (Figure 5D). ADG showed a significant positive correlation with fat, and a significant negative correlation with protein (*p* < 0.05). ADBH showed a highly significant positive correlation with fat (*p* < 0.01) and a significant negative correlation with protein and acidity (*p* < 0.05). ADCG showed a highly significant positive correlation with fat (*p* < 0.01) and a significant negative correlation with acidity (*p* < 0.05). Average daily body length gain (ADBL) and average daily cannon circumference gain (ADCC) did not show significant correlation with milk quality (*p* > 0.05).

### 3.6. The Relationship Between Lactating Period Microbiota and Calf Growth and Development

**Figure 6:**
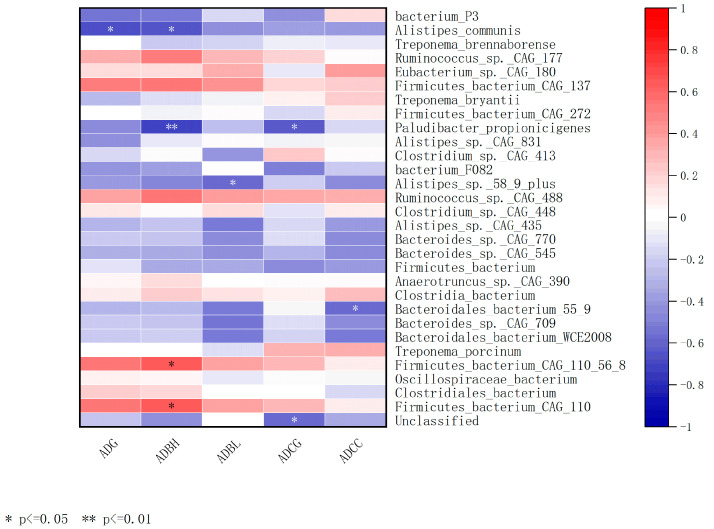
Heatmap of the correlation between the average daily growth of weight and body circumference and the gut microbiota.

At the species level (*p* < 0.05), ADG showed a significant negative correlation with *Alistipes_communis* (*p* < 0.05); ADBH showed a significant positive correlation with *Firmicutes_bacterium_CAG_110_56_8* and *Firmicutes_bacterium_CAG_110* (*p* < 0.05), a significant negative correlation with *Alistipes_communis* (*p* < 0.05), and a highly significant negative correlation with *Paludibacter_propionicigenes* (*p* < 0.01); ADBL showed a significant negative correlation with *Alistipes_sp._58_9_plus* (*p* < 0.05); ADCG showed a significant negative correlation with *Paludibacter_propionicigenes* (*p* < 0.05); and ADCC showed a significant negative correlation with *Bacteroidales_bacterium_55_9* (*p* < 0.05) (see Figure 6).

## 4. Discussion

### 4.1. Differences in Immune Indicators and Their Biological Significance

This study found significant differences in immune indicators between Pingliang red cattle and Simmental cattle during lactation, particularly in the levels of IL-6, IgG, and IgA. Pingliang red cattle exhibited higher levels of IL-6, while Simmental cattle showed significant advantages in the levels of antibodies such as IgA, IgG, IgM, and pro-inflammatory cytokines like IL-1β and TNFα. This difference may reflect distinct adaptive strategies in immune regulation between the two breeds, and this variation is closely related to their respective living environments and genetic backgrounds. As a pro-inflammatory cytokine, IL-6 plays a critical role in various physiological and pathological processes, including inflammation and immune regulation [26]. Elevated levels of IL-6 are commonly associated with acute immune responses and infection control. Particularly under environmental stress, a high expression of IL-6 can activate immune cells, thereby enhancing the host’s defense capacity [27]. Pingliang red cattle, living for an extended period in the relatively harsh environment of western China, may have developed a high adaptability to external stimuli. This is reflected in the rapid activation of immune responses through elevated IL-6 levels, enhancing their resistance to infections [28]. In contrast, the levels of immune factors such as IgA, IgG, IgM, IL-1β, and TNFα are significantly higher in Simmental cattle than in Pingliang red cattle. These immune factors play a crucial role in the host’s adaptive immune response [29,30]. The advantage of Simmental cattle in antibody (IgA, IgG, and IgM) levels suggests they may have stronger immune tolerance and higher immune memory capacity, which is consistent with their higher production performance and growth rate. Studies have shown that high levels of antibodies help animals effectively recognize and combat foreign pathogens, and this immune advantage is particularly beneficial in intensive farming environments, improving animal health and production performance [31,32]. These differences in immune markers may be closely related to the genetic backgrounds and environmental adaptability of the two breeds. Pingliang red cattle enhance their response to acute immune challenges through higher IL-6 levels, while Simmental cattle better cope with long-term pathogen stress by improving their antibody production and immune memory formation. Especially under intensive farming conditions, the higher levels of IgA, IgG, and IgM in Simmental cattle may be key factors in their adaptation to sustained immune pressure and pathogen invasion in their environment.

These differences may offer new strategies for livestock management. When facing environmental stress or pathogen challenges, selecting the appropriate breed based on its immune characteristics can maximize production potential. Future research should further explore the relationship between these immune traits, breed selection, feeding management, and environmental factors, with the aim of providing more precise scientific evidence to improve beef cattle health and production performance.

### 4.2. Comparison of the Composition and Function of Gut Microbiota

There are significant differences in the diversity and species composition of the gut microbiota between Pingliang red cattle and Simmental cattle. Although both breeds predominantly harbor *Firmicutes* and *Bacteroidetes* as major microbial phyla, the relative abundance of *Bacteroidetes* and *Fibrobacteres* is significantly higher in Pingliang red cattle compared to Simmental cattle, while the proportion of Actinobacteria is significantly higher in Simmental cattle. These differences not only reflect the diversity in the gut microbiota composition between the two cattle breeds, but also indicate significant functional differences in energy metabolism, fiber degradation, and immune regulation [33].

*Bacteroidetes* are closely associated with the degradation of dietary fiber and the production of short-chain fatty acids (SCFAs), particularly propionate and butyrate. These metabolites not only provide energy to the host but also have significant immune-regulatory effects [34]. *Fibrobacteres* are important cellulose-degrading bacteria in the rumen and intestines, involved in the breakdown of plant cell walls to produce short-chain fatty acids that can be absorbed by the host [35]. These results are consistent with the studies of Heredero [36] and Dunière [37], among others. This study points out that *Bacteroidetes* and *Fibrobacteres* play an important role in the fiber digestion of herbivores, especially in improving feed efficiency and energy acquisition. Pingliang red cattle are raised in a relatively open semi-pastoral environment, where the higher proportion of *Bacteroidetes* and *Fibrobacteres* may be aimed at better utilizing the fiber in coarse feed.

In contrast, the relative abundance of *Actinobacteria* in the intestines of Simmental cattle is significantly higher than that of Pingliang red cattle. *Actinobacteria* also play an important role in maintaining animal health. They can provide essential vitamins and amino acids, protect the intestinal barrier, acidify the gut microenvironment, inhibit the growth of pathogens, and reduce endotoxin production. This may be related to the characteristics of Simmental cattle, such as rapid growth and high production performance. The presence of these microorganisms may provide the host with more nutrients to support its growth and development. This finding is consistent with the results of Li [38], who also found that the abundance of Actinobacteria was positively correlated with the host’s growth performance. These differences in the gut microbiome composition have profound effects on the host’s physiological functions. First, the higher proportion of *Bacteroidetes* and *Fibrobacteres* in Pingliang red cattle enhances their efficiency in utilizing fibrous feed, thereby producing more short-chain fatty acids (SCFAs), such as propionate and butyrate, which not only provide energy to the host, but also play an important role in maintaining gut health and immune regulation. This enhanced fiber degradation capacity may make Pingliang red cattle better adapted to low-quality roughage, thus maintaining a better nutritional status and production performance in resource-limited environments.

This study conducted a differential analysis of the KEGG functional annotations and CAZy carbohydrate-active enzyme annotations of the gut microbiota in Pingliang red cattle and Simmental cattle, and found significant differences between the two cattle breeds in several metabolic pathways, indicating biological differences in metabolism and nutrient utilization capacity between the two breeds. Among them, carbohydrate metabolic pathways, such as galactose metabolism, were more enriched in Simmental cattle, suggesting that Simmental cattle may have stronger metabolic potential in carbohydrate metabolism and energy utilization, which may be related to their high production performance [39]. In addition, the differential enrichment of the ABC transporter family suggests that Pingliang red cattle may be more active in transmembrane transport, which is consistent with their adaptation to the nutritional demands in more challenging environments [40]. The differences in K07118 (aldehyde dehydrogenase) and K00346 (nitrate reductase) also indicate significant differences between the two cattle groups in nitrogen metabolism and aldehyde metabolism, which may be a result of their responses to environmental stress and differences in nutritional availability. The CAZy functional annotation analysis further reveals 31 differential metabolic pathways common to both Pingliang red cattle and Simmental cattle, including glycoside hydrolases (GHs), glycosyltransferases (GTs), polysaccharide lyases (PLs), carbohydrate-binding modules (CBMs), and carbohydrate esterases (CEs). The significant enrichment of carbohydrate-binding modules (CBMs) in Pingliang red cattle may indicate a unique functional advantage in the binding and degradation of carbohydrates such as cellulose and chitin, which aids in the better digestion and utilization of fiber under roughage conditions [41,42]. The differential enrichment of glycosyltransferases suggests that Pingliang red cattle have greater potential in carbohydrate synthesis and modification [43]. These differences in metabolic pathways may reflect the selective advantages in metabolic functions of the two cattle breeds in their long-term evolution and adaptation to different feeding conditions. The advantage of the microbiome in carbohydrate metabolism and energy acquisition in Simmental cattle may make them better suited to intensive feeding systems with high-nutrient feeds, whereas Pingliang red cattle’s advantage lies in their ability for material transmembrane transport and fiber utilization, which may help them better adapt to survival in low-nutrient, extensive conditions.

### 4.3. The Impact of Milk Quality on the Growth and Development of Calves

A comparative analysis of the milk composition during the lactation period of Pingliang red cattle and Simmental cattle highlighted distinct differences in key components such as milk protein, acidity, milk fat, and total solids content, reflecting the breeds’ unique physiological characteristics. Pingliang red cattle exhibited significantly higher milk protein content and acidity, which likely serve as adaptations to their environment in western China. The increased protein content provides essential nutrients for the calves’ early growth, supporting bone and muscle development, while higher acidity, potentially from organic acids like citric and lactic acid, may enhance the antimicrobial properties of the milk and promote gut health in the calves [44,45,46]. In contrast, Simmental cattle’s milk had significantly higher fat and total solids content, offering a more substantial energy source for rapid growth, especially in the early stages of life [47,48]. The higher milk fat content aligns with the breed’s breeding focus on reproductive and production performance, emphasizing energy transfer to calves to support their quick weight gain. Despite these differences in specific components, both breeds meet the basic nutritional needs of their calves, as indicated by their similar overall milk composition, which ensures proper growth and development during the lactation period.

### 4.4. Correlation Between Gut Microbiota, Immune Indicators, and Milk Quality

By analyzing the correlation between the gut microbiota of lactating cows and various immune indicators and milk quality, the results show significant associations between several gut microbial species and immune factors as well as milk components. These results provide new insights into how gut microbiota regulate host health and offspring development by influencing the immune system and milk quality. Multiple strains in the *Firmicutes* genus (such as *Firmicutes_bacterium_CAG_137* and *Firmicutes_bacterium_CAG_110_56_86*) were significantly positively correlated with immune factors such as IL-6, IgA, IgM, and TNFα (*p* < 0.05). IL-6 and TNFα are important pro-inflammatory cytokines, while IgA and IgM play key roles in mucosal immunity and humoral immunity. These positive correlations suggest that these strains may enhance the host’s immune response by promoting inflammatory responses and enhancing humoral immunity [49,50]. This finding is consistent with previous studies, which suggest that *Firmicutes* in the intestines of ruminants positively influence the activation of immune cells by metabolizing short-chain fatty acids and other metabolites [51,52]. In terms of milk composition, *bacterium_P3* and *Alistipes_communis* showed a significant positive correlation with acidity and protein, while *Alistipes_communis* also showed a significant negative correlation with urea and citric acid (*p* < 0.05). This suggests that the *Alistipes* genus may play an important role in regulating milk acidity, and its metabolic activity could influence the protein and urea content in milk. This phenomenon is also supported by some studies on ruminants, which indicate that gut microbes, through their metabolic products, directly or indirectly influence mammary gland metabolism and milk composition [53,54]. In addition, *Firmicutes_bacterium_CAG_137* showed a significant positive correlation with citric acid, while *bacterium_F082* showed a significant positive correlation with acidity and protein content, but a highly significant negative correlation with fat content. This relationship suggests that different strains may have distinct functions in the metabolism of various components in milk, and even exhibit competitive or inhibitory metabolic activities. This phenomenon may be related to the differentiated functions of these strains in energy metabolism and mammary gland metabolism. *Alistipes_sp._58_9_plus* showed a significant positive correlation with protein, but significant negative correlations with lactose, density, urea, and citric acid (*p* < 0.05). This complex relationship may reflect the multifunctional role of this strain in the metabolism of various milk components. *Alistipes* bacteria are generally considered microorganisms with diverse metabolic capabilities, able to utilize a variety of substrates and produce multiple metabolic products [55]. *Bacteroidales_bacterium_55_9* showed significant negative correlations with lactose, SNF, and urea, and extremely significant negative correlations with density and citric acid. This negative correlation may suggest that *Bacteroidales* bacteria influence the levels of these components in milk by metabolizing carbohydrates and proteins in the gut [56,57]. There are diverse and complex correlations between the gut microbiota of dairy cows and their milk quality and immune indicators. These correlations not only provide new evidence for understanding the impact of gut microbiota on milk quality and immune function, but also offer potential scientific basis for improving dairy cow health and production performance through the modulation of gut microbiota. Future research could further explore the causal relationship between these microbial communities and host metabolism, particularly by validating the specific roles of these microbes in milk quality and immune regulation through microbiota transplantation or nutritional interventions.

### 4.5. Correlation Between Milk Quality and Calf Growth and Development

In terms of calf growth, milk composition plays a crucial role, with both fat and protein levels significantly affecting nutrient intake and growth performance. Higher fat content in the milk is positively correlated with faster growth, particularly in terms of average daily gain (ADG) and body size growth [58]. This is consistent with studies showing that higher milk fat supports increased energy supply for rapid development; excessive protein, however, may negatively impact calf growth. Studies suggest that high protein levels can strain the digestive system of calves, leading to inefficiency in nutrient absorption and slower growth [59]. Similarly, higher milk acidity is negatively correlated with calf growth indicators, such as average daily body height (ADBH) and average daily body gain (ADCG), potentially due to its effect on gut health and nutrient availability [60]. This underscores the importance of optimizing milk composition to support calf growth, where adjusting fat levels for energy needs, and controlling protein and acidity, can enhance growth efficiency. Future studies should explore the long-term impact of these milk components on both calf health and production performance to refine feeding management strategies.

### 4.6. The Impact of the Maternal Gut Microbiota on the Growth of Calves

The gut microbiota of lactating cows plays a significant role in the growth and development of calves, particularly in the daily growth of various body size indicators, such as withers height, body length, and chest circumference. Overall, certain strains in the gut microbiota show different correlations with calf growth, including both positive and negative correlations, reflecting the potential influence of these microbes on host growth and development through various mechanisms. *Alistipes_communis* shows a significant negative correlation with both ADG and ADBH, suggesting that this strain may have an inhibitory effect on the overall growth of calves. *Alistipes* bacteria are generally considered symbiotic microbes in the intestines of humans and animals, but under certain conditions, they may negatively affect host growth by metabolizing specific substrates or producing particular metabolic products, such as short-chain fatty acids [61,62]. During the early developmental stage of calves, certain microbial metabolic products may have an inhibitory effect on the efficient utilization of energy and weight accumulation. Some strains in Firmicutes (e.g., *Firmicutes_bacterium_CAG_110_56_8* and *Firmicutes_bacterium_CAG_110*) are significantly positively correlated with ADBH, suggesting that these strains may promote the growth of calf height. This may be related to the important role of Firmicutes in energy metabolism and cellulose degradation, as they are capable of producing beneficial metabolic products such as short-chain fatty acids, which provide energy to the host and promote skeletal growth and development [63,64]. This finding is consistent with previous research, which suggests that *Firmicutes* have a positive impact on energy metabolism and growth in the gastrointestinal tract of ruminants [65,66]. It is worth noting that *Paludibacter_propionicigenes* is significantly or highly significantly negatively correlated with both ADBH and ADCG, suggesting that this strain may inhibit the growth of withers height and chest girth in calves to some extent. This negative correlation may be related to the metabolic characteristics of the strain, such as its production of certain organic acids, which may negatively affect the host’s metabolism and inhibit the growth of the calves’ body measurements [67]. Furthermore, *Alistipes_sp._58_9_plus* and *Bacteroidales_bacterium_55_9* are significantly negatively correlated with ADBL and ADCC, respectively. These results further suggest that different microbial strains have complex effects on various growth parameters of calves, potentially related to their metabolic activities in the gut, nutrient utilization efficiency, and the manner in which they regulate host metabolism. *Bacteroidales* bacteria play an important role in the degradation of cellulose and other complex carbohydrates, and their metabolic products may inhibit the growth of bones and connective tissues [68,69].

Our research suggest that some microbial strains in the gut of lactating cows play a significant regulatory role in the growth and development of calves, with both potential promoting and growth-inhibiting effects. Understanding the impact of these microbes on host growth and development is crucial for developing scientifically based feeding management strategies. Future research should further explore how these microbes influence host growth by regulating metabolic pathways, and investigate how microbial interventions (such as probiotics or microbiome transplantation) can be used to optimize calf growth and development.

## 5. Conclusions

This study provides a comprehensive analysis of the immune indicators, gut microbiome composition, and milk quality in relation to calf growth and development in Pingliang red cattle and Simmental cattle during the lactation period. The main findings are as follows: First, significant differences were observed in immune indicators between the two breeds. Pingliang red cattle exhibited higher levels of IL-6, whereas Simmental cattle showed greater concentrations of IgG and IgA. These differences suggest distinct immune regulation strategies between the two breeds. Second, the gut microbiome composition varied between the breeds. Pingliang red cattle had a higher abundance of *Bacteroidetes* and *Fibrobacteres*, which are involved in cellulose degradation and short-chain fatty acid production. In contrast, Simmental cattle had more *Actinobacteria*, which may be linked to their faster growth and B-vitamin synthesis. Third, milk fat content was positively correlated with calf weight and body size, highlighting its role in providing energy and supporting immune function. The relationship between milk protein content and calf growth was more complex, with excessive protein potentially limiting weight gain. Finally, the mother cow’s gut microbiome influenced the calf’s microbiome colonization and growth, with milk-derived nutrients and metabolites playing a key role. This vertical transmission of the microbiome contributes to improved calf health and development. In conclusion, maternal gut microbiome, immune regulation, and milk quality significantly impact calf growth, health, and development. Future studies should explore how the cow’s microbiome in different reproductive stages affects calf health, providing valuable insights for enhancing livestock management and animal health.

## Data Availability

Follow-up research on this project is ongoing; please contact the corresponding authors with reasonable requests. All data covered in the article are available from the authors.

## Supplementary Materials

The following supporting information can be downloaded at: https://www.mdpi.com/article/10.3390/ani15020197/s1, Table S1: Differences in the KEGG functional annotation of the gut microbiota between two breeds of lactating cows; Table S2: Differences in the CAZy functional annotation of the gut microbiota between two breeds of lactating cows.
